# Gender Differences in Academic Resilience and Well-Being among Senior High School Students in Ghana: A Cross-Sectional Analysis

**DOI:** 10.3390/children11050512

**Published:** 2024-04-24

**Authors:** Mustapha Amoadu, Edmond Kwesi Agormedah, Paul Obeng, Medina Srem-Sai, John Elvis Hagan, Thomas Schack

**Affiliations:** 1 Department of Health, Physical Education and Recreation, University of Cape Coast, Cape Coast CC 3321, Ghana; mustapha.amoadu001@stu.ucc.edu.gh (M.A.); paul.obeng@stu.ucc.edu.gh (P.O.); 2 Department of Business & Social Sciences Education, University of Cape Coast, Cape Coast CC 3321, Ghana; edmond.agormedah@ucc.edu.gh; 3 Department of Health, Physical Education, Recreation and Sports, University of Education, Winneb P.O. Box 25a, Ghana; mssai@uew.edu.gh; 4 Neurocognition and Action-Biomechanics-Research Group, Faculty of Psychology and Sports Science, Bielefeld University, Postfach 10 01 31, 33501 Bielefeld, Germany; thomas.schack@uni-bielefeld.de

**Keywords:** Ghana, senior high school, interventions, mental health, resilience, well-being

## Abstract

Senior high school (SHS) students are at risk of stress and other adverse exposures that may negatively affect their well-being and possibly cause attrition. The concepts of academic resilience and well-being share commonality as psychological attributes linked to positive functioning among students. Despite this connection, there seems to be limited research exploring these concepts across genders among SHS students in developing regions. This study examined the gender difference in academic resilience and well-being among SHS students in Ghana. Using a cross-sectional survey design, 190 SHS students in three schools from Kwahu North and South district (i.e., Afram Plains) of Ghana’s Eastern Region completed the Academic Resilience Scale (ARS-30) and College Student Subjective Wellbeing Questionnaire (CSSWQ). The sample consists of 102 males and 88 females, with a mean age of 17.83 years. The data were analyzed using independent samples *t*-tests and hierarchical regression. The study established that students have a moderate level of academic resilience and a higher level of well-being, with no statistically significant variation in students’ academic resilience (*t* = 0.718; *p* = 0.474) or well-being (*t* = −1.596; *p* = 0.112) across gender. Further, the study discovered that resilience significantly predicted academic well-being (*B* = 0.425; *SE* = 0.050; *t* = 8.50; *p* < 0.001). This study highlights the importance of promoting gender-sensitive intervention strategies that enhance the academic resilience and well-being of SHS students and help boost their educational attainment.

## 1. Introduction

Well-being is essential for academic success and overall happiness among students. Academic well-being encompasses a student’s overall satisfaction and positive emotions related to their academic experiences. However, students often experience challenges because they have to attend to their academic, physical, mental, and social needs simultaneously [1,2]. These challenges might negatively affect their academic well-being and other behavioral outcomes. In the school environment, students face many new and difficult school activities every day in the classroom, and they are expected to cope with various pressures and expectations [1,2]. For students, one of the most important factors taken from the human adaptive system to help them overcome challenges and promote their maximum growth and well-being is resilience, a psychological construct considered to differentiate an individual’s success and failure in the face of life adversity [3,4,5]. This construct is associated with good adaptation to threats and problems [6] or the ability to recover from life’s problems [7].

In the framework of SHS education, students must demonstrate resilience to academic challenges, known as academic resilience [2]. This relates to a student’s ability to overcome academic challenges, adapt to adversity, and maintain a positive quality of life [8]. Wang and Gordon [9] believe that academically active students (i.e., learners with academic resilience) can turn academic situations into a source of motivation by maintaining a positive attitude, re-positioning themselves with clear goals, and developing exceptional problem-solving skills. This plays an important role in ensuring the success and sustainability of education [10].

Research on academic resilience and well-being has gained popularity in school settings due to its association with positive international achievement and school-related adjustment [11,12,13]. Existing investigations have established the relationship between academic resilience and well-being in multiple bio-psychosocial contexts, including contextual determinants such as gender, age, socio-economic status, ethnic group, and environmental stressors [14,15,16,17,18]. Globally, many researchers have found differences in academic resilience and academic achievement between boys and girls [10,19], with contrasting results. For instance, Erdogan et al. [20] in Turkey and Sarwar et al. [21] in Pakistan observed higher resilience in males, while Latif and Amirullah [22] found no significant difference in academic resilience among males and females. In Africa, Mwangi and Ireri [10] in Kenya and Olaseni [23] in Nigeria highlight greater resilience among females. Erdogan et al. [20] advocate for further investigation into the impact of gender on academic resilience. These studies have highlighted the importance of gender as a key factor influencing people’s resilience and vulnerability [24]. These differences may be reflected in coping strategies, persistence, and the overall ability to recover from academic difficulties [8].

In Ghana, discrepancies in academic resilience and well-being continue among SHS students [25]. Conversely, conflicting results show a moderate effect of gender on academic resilience, with some scholars indicating higher resilience in female students while others suggest that male students have higher academic resilience [10,20]. The persistence of gender educational gaps in Ghana, similar to findings in Kenya [10], led to an in-depth study of the difficulties underlying educational resilience. Because gender dynamics are linked to family and cultural factors, it is important to understand the unique ways that male and female students navigate academic challenges.

Although academic resilience and well-being are important personal psychological resources in meeting academic challenges, there is an emerging and increasing attention to understanding how these elements contrast across sexual orientations (gender) in the SHS setting, particularly in developing nations [26,27]. The justification for this call is that little research has been carried out in this area, and some of the current research does not focus on SHS students but rather higher education [14,16]. There is a gap to analyze and explain the discrepancies in academic resilience and well-being between male and female SHS students [26,27]. In spite of the developing body of examinations on academic resilience and well-being, the exact gender differences among SHS students in Ghana have received little attention.

Accordingly, assessing gender differences in academic resilience and well-being among SHS students in Ghana embraces urgent ramifications for education equity, strategy planning, policy formulation, cultural and societal advancement [28]. By unveiling these nuances, policymakers can make designated interventions to help the two sexes, enhancing academic performance and overall psychological well-being. This inquiry, employing a sample of students within the Ghanaian context, tries to unveil the intricate relationship between academic resilience and well-being as well as gender differences in these two constructs. Through a thorough investigation, this examination intends to contribute significant insights to the international discourse while addressing the unique socio-cultural/contextual dynamics shaping students’ educational journeys in Ghana.

Therefore, the rationale of the present study is two-fold. First, it examined the gender difference in academic resilience and well-being among SHS students in Ghana. Second, this study examined the relationship between academic resilience and psychological well-being of the target sample. It was hypothesized that male and female SHS students would significantly differ in their academic resilience and well-being scores. Additionally, a significant relationship would exist between academic resilience and academic well-being among the SHS students.

### Theoretical Underpinning

This current study is rooted in the social role theory (SRT). The SRT posits that gender differences in behavior and attitudes can be attributed to societal expectations and norms associated with gender roles [29]. This model suggests that societal expectations and norms about gender roles significantly influence people’s behavior and shape their knowledge, skills, and practices in different settings, including education [29]. The SRT offers an important perspective for examining gender differences in the academic resilience and well-being of SHS students in Ghana [29]. According to this theory, gender differences in academic resilience and academic well-being may be influenced by the roles and expectations placed on boys and girls in society. According to this theory, men and women are socialized from a young age to conform to certain gender-specific roles and norms, which can influence their approach to academic challenges [29]. Therefore, it is important to understand the social beliefs that affect the actions of boys and girls so as to explain their academic resilience and well-being. In the context of academic resilience, gender differences can be attributed to the different socialization experiences that males and females undergo. The SRT suggests that girls may face additional barriers to academic resilience due to societal stereotypes about their abilities in certain subjects, such as math and science. These stereotypes may lead to a lack of confidence and motivation in girls, impacting their resilience in these academic areas. Moreover, the SRT suggests that boys may experience higher levels of academic well-being due to the emphasis placed on success and achievement in male gender roles. Boys may have a greater sense of confidence and self-worth related to their academic abilities, leading to higher levels of academic well-being.

In addition, the way families, schools, and communities socialize SHS students based on gender may influence their patterns of academic resilience and well-being disparities. Eagly [29] suggests that societal perceptions of men’s and women’s career paths influence differences in academic motivation, resilience, and overall well-being. Ijadi-Maqsudi et al. [24] highlight gender as a key factor influencing human resilience and vulnerability. According to Eagly’s SRT [29], social expectations and norms about gender roles strongly influence a person’s behavior and experiences, including his/her approach to challenges. Consequently, examining the link between academic resilience and well-being in the context of gender dynamics is consistent with empirical evidence and the SRT. Thus, the choice of the SRT as the theoretical framework aligns with the study’s objectives by providing a comprehensive perspective on how societal expectations and norms surrounding gender roles influence academic resilience and well-being among SHS students in Ghana. SRT emphasizes the impact of societal beliefs and gender roles on individuals’ behaviors and experiences, particularly in educational settings. By examining how societal perceptions shape students’ responses to academic challenges and disparities in resilience and well-being, this study contributes to a deeper understanding of gender dynamics in academic contexts, consistent with the principles of the SRT.

## 2. Material and Methods

### 2.1. Study Context

Public senior high schools (SHS) in the Kwahu North and South district (i.e., Afram Plains) of the Eastern Region of Ghana were the focus of this research because of the unique characteristics of the populace. The World Bank report [30] noted that this part of Ghana is a poverty-prone area characterized by a higher rate of school drop-outs, with the populace having a limited chance of breaking out of poverty. Hence, this study focused on in-school adolescents from this setting, which served as a hub for the data collection. This region, marked by poverty and higher rates of school dropouts, underscores the importance of understanding factors that can potentially mitigate educational challenges. Gender differences may intersect with socio-economic disparities, influencing how students navigate academic hurdles. Furthermore, assessing the role of academic resilience in promoting well-being is crucial for developing targeted interventions that can uplift students in this disadvantaged setting, potentially breaking the cycle of poverty through improved educational outcomes. The duration of this transitional stage of school education takes 3 years after their basic education [31]. This cohort of students was chosen due to their developmental stage, which poses unique challenges related to academics, living standards, and personal, social, and psychological needs [32,33].

### 2.2. Study Design and Participant Selection

The cross-sectional survey sampled 190 SHS students from the Kwahu North and South district (i.e., Afram Plains) of Ghana. A stratified sampling method was utilized. The districts were demarcated into 2 zones as strata for the study. Given this categorization, three SHS within the zones were randomly selected using the fishbowl approach. Within the schools, systematic sampling techniques were used to select individual students. Due to the low school enrollment in the target areas, approximately 370 students were earmarked. However, only 190 provided valid responses, representing a 51.35% response rate, understandably because all the sampled schools were conducting examinations at the time of the data collection. The low response rate of 51.35% in the data collection process could be attributed to scheduling conflicts during examination periods and limited access to day students who do not reside on school premises. These challenges hindered the participation of students, resulting in a reduced number of valid responses collected compared to the initially earmarked student population of approximately 370. Among them, 53.7% were boys, and 44.3% were girls. On average, participants were around 17.83 years old, with a typical age range of about 2 years. In terms of where they lived, 42.1% resided in cities, while 57.9% lived in rural areas. Looking at their school grades, 12.6% were in the first year (SHS 1), 53.7% were in the second year (SHS 2), and 33.7% were in the third year (SHS 3). This shows a diverse representation across different stages of SHS education in this geographical area (see Table 1).

The eligibility criteria included (1) informed consent provided by students above 18 years old, and those who were below 18 years old required parental consent; and (2) participants must be officially enrolled in the sampled schools.

### 2.3. Study Measures

#### 2.3.1. Academic Resilience

The academic resilience of participants was measured using Cassidy’s [14] 30-item Academic Resilience Scale (ARS-30). The survey instrument has 3 main subscales: perseverance (14 items; e.g., “I would not accept the teachers’ feedback in school”), reflecting and adaptive help-seeking (9 items; e.g., “I would use my past successes to help motivate myself in school”), and negative affect and emotional response (7 items; e.g., I would probably get annoyed in school”). For each item, participants were asked to indicate the extent to which they agreed or disagreed with it on a 5-point Likert-type scale that ranged from 1 (SD—strongly disagree), 2 (D—disagree), 3 (N—neither disagree/agree), 4 (A—agree), and 5 (SA—strongly agree). The scoring was computed by summing all scores obtained on each item. For easy interpretation of the mean score, a mean criterion was established based on the five-point Likert scale using the scoring guide of Cassidy [14]. A mean score between 30.00 and 69/60 was considered a low level of academic resilience, while a mean score of 69.90–109.80 and 110.10–150.00 was treated as a moderate and high level of academic resilience, respectively. The reliability coefficient value of 0.70 obtained for this scale was deemed acceptable [34,35,36].

#### 2.3.2. Academic Well-Being

The College Student Subjective Wellbeing Questionnaire (CSSWQ) [37] was used to measure the academic well-being of SHS students. The CSSWQ is a 16-item self-report and evidence-based rating scale with four subscales, including academic satisfaction (4 items; e.g., “I have had a great academic experience at this school”), academic efficacy (4 items; e.g., “I am a hard worker in my classes”), school connectedness (4 items; e.g., “I feel like a real part of this school”), and college gratitude (4 items; e.g., “I am so thankful that I am getting a secondary school education”). For each item, participants were asked to indicate the extent to which they agreed or disagreed with it on a 5-point Likert-type scale that ranged from 1 (SD—strongly disagree), 2 (D; disagree), 3 (N—neither disagree/agree), 4 (A—agree), and 5 (SA—strongly agree). The scoring was computed by summing all scores obtained on each item. To determine the level of academic well-being, a mean criterion was used based on the scoring guide of the scale. A mean score between 16.00–37.12, 37.28–58.56, and 58.72–80.00 were considered to be low, moderate, and high levels of academic well-being, respectively. The reliability coefficient value obtained for this scale was 0.84, and it is deemed reliable for the sample.

#### 2.3.3. Data Collection Procedure

The data collection process adhered to a structured protocol in line with ethical guidelines and regulations, beginning with the procurement of formal approval from both the district education office and the headmasters of the SHS involved. Ethical clearance was obtained from the Institutional Review Board of the University of Education Winneba, with reference number: DAA/P.1/Vol.1/39. All standard procedures in accordance with relevant guidelines and regulations under the 6th edition of the Declaration of Helsinki were adhered to. Following this, the researchers initiated familiarization meetings with school authorities to elucidate this study’s rationale and objectives. Subsequently, all students and teachers convened in the school auditorium for a comprehensive briefing, during which the survey instrument’s content and purpose were elucidated. Ethical considerations, such as voluntary participation, confidentiality, and anonymity, were underscored, and participants were assured that their data would be used solely for research purposes. The participants were informed about their rights to withdraw from the study without any penalty. The consent form was given to each student to read and sign. For students under the age of 18, the school headteachers (i.e., principals) served as their proxy parents. Each participant provided individual consent before receiving the survey instrument, which was administered with the assistance of teachers. The data collection spanned a period of 2 weeks in August 2023, with questionnaires administered within 15–20 min each. Upon completion, all filled-out questionnaires were collected by teachers and securely sealed in brown envelopes to maintain confidentiality.

### 2.4. Data Analysis

Descriptive and correlational analyses were conducted to establish the potential relationship between academic resilience and well-being. Furthermore, hierarchical multiple regression predicted SHS students’ academic well-being. Socio-demographic variables (sex, residential status, age, and grade level) were first entered into the hierarchical multiple regression (Model 1). Academic resilience was then entered into the second model (Model 2). Hierarchical multiple regression was chosen for its ability to systematically assess the unique contribution of different sets of variables to the prediction of an outcome variable. By entering socio-demographic variables such as sex, residential status, age, and grade level in the first model and subsequently adding academic resilience in the second model, this approach allowed for the examination of how much variance in academic well-being could be accounted for by each set of variables. Additionally, hierarchical multiple regression enables the determination of whether the inclusion of academic resilience as a predictor adds significant explanatory power beyond socio-demographic factors alone, providing valuable insights into the unique contribution of academic resilience to SHS students’ well-being. In addition, an independent *t*-test was conducted to compare male and female SHS students on academic resilience and academic well-being. Compliance with the minimum parameters of each statistical test was analyzed. Data management was conducted using IBM Statistical Package for Social Sciences version 27.

## 3. Results

### 3.1. Preliminary Analyses

The analysis exploring correlations between the variables unveiled noteworthy associations. Academic resilience exhibited a significant positive correlation with academic well-being (r = 0.523, *p* < 0.001), suggesting that higher levels of resilience were linked to higher levels of academic well-being. See Table 2 for more details on our correlational analysis.

### 3.2. Comparison between Male and Female SHS Students

Table 3 presents an analysis of an independent *t*-test that compares male and female students on academic resilience and well-being. No statistical difference was detected between male and female students based on their academic resilience (*t* = 0.718, *p* = 0.474) and well-being (*t* = −1.596, *p* = 0.112) scores.

### 3.3. Hierarchical Linear Regression Analysis of Predictors of Academic Well-Being

Table 4 presents the results of the hierarchical linear regression analyses. The assumptions of regression were examined using the recommendations of Osborne and Waters [38] and Williams et al. [39]. Partial plots and scatter plots of the predictors in the model confirmed that the assumptions of linearity and homoscedasticity were not violated. Also, high tolerance values > 0.1 and variance inflation factor (VIF) values <10 were found, which further indicated that issues of multicollinearity do not exist among the variables in the regression model [38]. Also, with a Durbin–Watson statistic of 1.723, it is assumed that issues of autocorrelation do not exist in the data. We detected no violation of the normality of the residual distribution assumption. The results indicated the existence of an association between academic resilience and academic well-being, as reported by the students. In Model 1, age, sex, residential status, and grade level explained 8% (adjusted R square of 0.080) of the variance in academic well-being. The addition of academic resilience to the model (Model 2) explained an additional 26% variance (change in adjusted R square of 0.256) in academic well-being. Thus, in Model 2, the variables explained 31.8% of the variance in academic well-being, which indicates a small predictive power of the regression model. Additionally, there was a low mean square error (MSE) of 0.002, which indicates that the model’s prediction has a relatively low error.

## 4. Discussion

The academic well-being of students is a critical factor for student academic and behavioral outcomes and an indicator for achieving Sustainable Development Goal 4 (SDG 4). It is recognized that students’ internal resources, like academic resilience, are a significant contributor to their well-being in schools. This inquiry examines gender differences in students’ academic resilience and well-being, as well as the association between academic resilience and well-being among SHS students.

Concerning the first objective, it was discovered that the students have a high level of academic well-being, a finding that lends support to the study of Khan et al. [40]. This outcome is an indication that the students are experiencing high physical, mental, and emotional health within the educational system. This may significantly impact their ability to thrive academically. When students are physically and mentally well, they are more likely to concentrate, participate actively, and retain information. When students feel supported, safe, and healthy, they are more likely to engage in learning and achieve positive educational outcomes. There was a non-statistically significant difference in the academic well-being of students based on gender. The female students demonstrated higher academic well-being than the male students but did not meet statistical significance. This result implies that academic well-being is not sensitive to the gender of students. The findings of the current investigation contradict previous studies that have established that female students have higher academic well-being than male students [41,42]. Conversely, the finding disagrees with other studies due to perhaps sample characteristics and contextual issues [43,44]. For example, Marquez [44] revealed that female students reported lower academic life satisfaction (an aspect of academic well-being) than male students. The inconsistencies in results could be explained by societal expectations, self-esteem, and access to resources by both male and female students. These factors may influence how individuals experience academic well-being differently based on their gender. Drawing from the SRT, it is argued that both male and female students experienced equal social expectations and norms associated with gender roles. It is also important to consider the intersectionality of gender with other identities, such as age, race, ethnicity, sexuality, and socioeconomic status, as these factors can further impact academic well-being.

We further discovered that the students have a moderate level of academic resilience. The moderate level of academic resilience among students is an indication that they have a reasonable internal ability (resource) to overcome challenges, adapt to changes, and thrive academically, even in difficult circumstances. These students are likely to be better equipped to achieve their educational goals while maintaining their mental and emotional health. They may engage actively in their learning, face challenges with confidence, and persist in their studies. These attempts may lead to enhanced learning outcomes and a higher-quality educational experience. Similar to the study of Latif and Amirullah [22], there was a non-statistically significant difference in the academic resilience of students across genders. This result implies that academic resilience is not sensitive to the gender of students. Thus, there were no significant differences in how males and females respond to academic challenges, stress, and setbacks. Though male students demonstrated a moderate level of academic resilience compared to female students, this difference did not meet statistical significance. Globally, differences exist in the academic resilience of boys and girls [10,19]. Extant researchers have found higher resilience in males [20,21], which is contrary to current findings, although greater resilience among females has been observed by some scholars [10,23].

The absence of a statistical difference between male and female students in terms of academic resilience and well-being in the Ghanaian context may be attributed to various factors that interact within the local educational landscape. One plausible explanation could be the evolving societal expectations and educational norms in Ghana that aim for more equitable opportunities and treatment for both genders. Drawing from the SRT, it is argued that both male and female students experienced equal social expectations and norms associated with gender roles. Thus, both males and females may exhibit internalizing and externalizing behaviors in response to academic challenges. Over time, efforts to promote gender equality in education may have contributed to a more level playing field, diminishing the traditional disparities that might have existed. Cultural contexts in Ghana often emphasize the importance of education for both male and female individuals, promoting a shared commitment to academic success. As a result, societal attitudes and expectations regarding academic achievement may have become more uniform across genders, leading to similar levels of academic resilience and well-being among male and female students [19]. Moreover, the Ghanaian educational system’s emphasis on inclusivity and holistic support for all students may have played a role. Support structures within schools, such as counseling services and mentorship programs, might be designed to address the diverse needs of students, regardless of gender [25]. This approach could foster a sense of inclusiveness and support for both male and female students, contributing to comparable levels of academic resilience and well-being.

Further, Ghanaian cultural values may encourage a collective approach to problem-solving and resilience. Both male and female students might be exposed to similar societal expectations regarding perseverance and determination in the face of academic challenges. This shared cultural ethos could lead to parallel coping mechanisms and responses to academic stressors, contributing to the observed lack of statistical difference. It is essential to recognize that gender intersects with other identity factors in the Ghanaian context, such as socioeconomic status and regional and ethnic diversity. These intersections may have further contributed to a nuanced and complex interplay of factors that result in similar academic resilience and well-being scores for male and female students. To maintain and enhance this equality, educators and policymakers should continue to foster a supportive and inclusive learning environment that considers the diverse experiences and needs of all students.

Using the hierarchical regression model, we discovered that the demographic profiles (age, sex, residential status, and grade level) of the students explained 8% of the variance in academic well-being, while academic resilience explained 26% of the variance in academic well-being after controlling for demographic profiles. Students’ academic resilience significantly predicted their academic well-being. The observed results from the hierarchical regression model reveal insightful contextual reasons for the relationship between academic resilience and well-being among students. In the Ghanaian educational context, where students often face multifaceted challenges such as academic pressures, societal expectations, and potential disruptions related to residential status and grade levels [25], the limited explanatory power (8%) of demographic profiles underscores the nuanced nature of these challenges. On the other hand, the substantial 26% variance explained by academic resilience suggests that students’ ability to bounce back from setbacks, cope with stress, and maintain a positive outlook significantly influences their overall well-being [45]. This psychological index may be particularly relevant in a setting where the educational landscape demands adaptability and perseverance. The findings imply that, irrespective of demographic factors, fostering academic resilience becomes a pivotal strategy for promoting students’ well-being, offering practical insights for educators, policymakers, and support services in crafting interventions tailored to enhance students’ psychological and academic thriving. Additionally, this result implies that academic resilience contributes to students’ overall well-being. This link may help them develop a positive mindset, manage stress effectively, and maintain a healthy work–life balance. By prioritizing students’ well-being, we are not only supporting their academic success but also nurturing their holistic development. These results extend current literature in that a higher level of resilience is related to more positive psychological well-being [3,45]. Moreover, resilience largely and positively contributed to psychological well-being [16,27,46,47]. The results are comparable to those of Idris et al. [3], who found that resilience explained 48.2% of the variance in psychological well-being.

### 4.1. Strengths and Limitations

To the best of our knowledge, this is the first study to explore the association between academic resilience and the academic well-being of SHS students in Ghana. This study, therefore, provides useful information for further research. As the current study is relational, causality cannot be implied. Therefore, interpretations of the findings should be made cautiously. The survey approach used may introduce some elements of bias, which is generally likely to affect the results. One limitation of this study stemming from the low response rate is the potential for non-response bias. The fact that only 51.35% of the targeted students provided valid responses could introduce a bias if those who did not respond differ systematically from those who did. This could affect the generalizability of the findings and may limit the extent to which the results can be applied to the broader population of students in the target areas. Because of the cross-sectional nature of the present study, it is difficult to draw conclusions based on its long-term effect on the identified patterns. One limitation of this study is that certain school characteristics, such as the type of school, prestige, or presence of special programs aimed at supporting students’ academic well-being, were not included in the analysis, which may have an effect on the findings. Conducting future research on these thematic areas with longitudinal designs might be very useful in identifying the unfolding psychological reactions to guide appropriate interventions. Future research could consider employing multilevel modeling to control for these factors and further explore their impact on academic resilience and well-being among SHS students in Ghana using a larger sample size.

### 4.2. Implications and Future Directions

The findings of this study have noteworthy practical implications for various stakeholders within the Ghanaian educational landscape. The revelation of a high level of academic well-being among students, indicating positive physical, mental, and emotional health within the educational system, underscores the necessity for sustained efforts to foster supportive and inclusive learning environments. The recognition of gender differences in academic well-being, with female students exhibiting higher levels, calls for targeted interventions and initiatives tailored to address the distinct needs and challenges faced by both male and female students. This highlights the urgency for specific programs promoting mental and emotional well-being, particularly for male students who may experience comparable stressors but may not overtly express them. Furthermore, the non-significant difference in academic resilience between male and female students’ challenges prevailing global trends and underscores the potential success of gender-inclusive educational policies and cultural values in Ghana. The shared moderate level of academic resilience indicates a collective approach to problem-solving and perseverance, urging educational institutions and policymakers to fortify support structures that encourage resilience and determination, thereby contributing to the overall well-being of students.

The results from the hierarchical regression model, emphasizing the substantial contribution of academic resilience to academic well-being, suggest practical strategies for enhancing students’ psychological and academic thriving. It advocates for prioritizing the development of programs and interventions that specifically target the enhancement of academic resilience skills among students. By equipping students with the ability to rebound from setbacks, cope with stress effectively, and maintain a positive mindset, educational institutions can cultivate an environment conducive to holistic student development. Moreover, the intersectionality of gender with other identity factors, such as ethnicity, socioeconomic status, and regional diversity, calls for a comprehensive and nuanced understanding of students’ experiences. Implementing intersectional approaches in educational policies and support services will contribute to a more tailored and effective response to the diverse needs of students.

In considering future research directions, it is pertinent to explore the potential benefits of adopting longitudinal study designs to further elucidate the dynamics of academic resilience and well-being among Ghanaian university students. Longitudinal studies would enable researchers to track changes in these constructs over time, offering valuable insights into how various factors influence students’ psychological well-being as they progress through their academic journey. Additionally, there is a pressing need to investigate the disparities that exist between rural and urban school students in terms of academic resilience and well-being. Such investigations could shed light on the unique challenges and strengths associated with different socio-environmental contexts, informing targeted interventions and policies aimed at promoting student success across diverse settings. Therefore, future research endeavors should consider incorporating longitudinal methodologies and exploring rural–urban differentials to advance our understanding of academic resilience and well-being among Ghanaian university students.

To enhance academic resilience and well-being among Ghanaian students, it is imperative to develop integrated resilience training programs within educational curricula, providing students with coping skills and stress management techniques. Additionally, expanding access to mental health support services tailored to address gender-specific concerns and cultural sensitivities is crucial. Establishing gender-inclusive support initiatives, fostering community engagement, and conducting longitudinal research to assess intervention effectiveness are essential steps. Furthermore, analyzing disparities between rural and urban school students will inform region-specific interventions. These efforts will create a supportive environment conducive to holistic student development, promoting academic resilience and well-being across diverse backgrounds. In order to comprehensively address gender-related barriers and biases in academia, it is imperative to reinforce the call for collaborative efforts among stakeholders, including educational institutions, policymakers, community organizations, and relevant stakeholders, to implement targeted interventions and initiatives aimed at promoting gender equity and inclusivity within the educational landscape.

## 5. Conclusions

This current investigation discovered that SHS students have a moderate level of academic resilience and a high level of academic well-being. There was no statistically significant difference in academic resilience or academic well-being based on gender. Students’ academic resilience significantly and positively predicted their academic well-being. The findings highlight the importance of promoting academic resilience and academic well-being to enhance educational attainment. Educational institutions are encouraged to continually create an inclusive atmosphere that supports diversity and ensures equal educational opportunities for all. Collaboration among policymakers, educators, parents, and communities is crucial to creating an environment that prioritizes the well-being of learners. It is crucial for educational institutions and policymakers to continue addressing gender-related barriers and biases in order to foster inclusive and supportive environments for all students. Implementing measures such as gender-responsive teaching practices, mentorship programs, support networks, and challenging gender stereotypes can contribute to promoting academic resilience and well-being among students of all genders.

## Figures and Tables

**Table 1:** The socio-demographic background of study participants (*n* = 190).

Variable	f (%)	M (SD)
Gender		
Male	102 (53.70)	
Female	88 (44.30)	
Age		17.83 (2.00)
Residential status		
Urban	80 (42.10)	
Rural	110 (57.90)	
Grade level		
SHS 1	24 (12.60)	
SHS 2	102 (53.70)	
SHS 3	64 (33.70)	

**Table 2:** Correlation (Pearson r) between study variables.

	Variables	1	2	3
1	Age (years)	-		
2	Academic resilience	−0.137	-	
3	Academic well-being	−0.256 ***	0.523 ***	-

*** Correlation is significant at the 0.001 level (two-tailed).

**Table 3:** Differences in students’ academic resilience and well-being.

Factor	Male		Female		*t*-Value	Sig
	Mean	SD	Mean	SD		
Academic well-being	63.177	9.430	65.284	8.650	−1.596	0.112
Academic resilience	107.265	11.671	106.114	10.203	0.718	0.474

**Table 4:** Hierarchical multiple linear regression coefficients of predictors of academic well-being.

Model	Variable	R^2^_adj_	∆R^2^_adj_	*B*	SE	*Beta*	Sig.	Tol	VIF
1	(Constant)	0.060	0.080	80.712	6.305		0.000		
	Gender			1.655	1.304	0.091	0.206	0.972	1.029
	Age			−1.158	0.370	−0.254	0.002	0.753	1.327
	Residential status			1.237	1.323	0.067	0.351	0.963	1.039
	Grade level			−0.130	1.134	−0.009	0.909	0.762	1.312
2	(Constant)	0.318	0.256	28.186	8.234		0.000		
	Gender			2.111	1.112	0.116	0.059	0.969	1.032
	Age			−0.865	0.317	−0.190	0.007	0.744	1.343
	Residential status			1.931	1.130	0.105	0.089	0.957	1.044
	Grade level			−0.042	0.967	−0.003	0.965	0.762	1.312
	Academic resilience			0.425	0.050	0.513	0.000	0.972	1.028

Note: R^2^_adj_, adjusted R square; ∆R^2^_adj_, R square change. *B*, unstandardized coefficient beta; and Tol, tolerance; VIF, variance inflation factor. Dependable variable: academic well-being.

## Data Availability

The data presented in this study are available on request from the corresponding author. The data are not publicly available due to privacy restrictions.
